# Associations between phenotypic characteristics and clinical parameters of broilers and intestinal microbial development throughout a production cycle: A field study

**DOI:** 10.1002/mbo3.1114

**Published:** 2020-10-17

**Authors:** Jannigje G. Kers, Jean E. de Oliveira, Egil A. J. Fischer, Monique H. G. Tersteeg‐Zijderveld, Prokopis Konstanti, Jan Arend (Arjan) Stegeman, Hauke Smidt, Francisca C. Velkers

**Affiliations:** ^1^ Department of Population Health Sciences Division of Farm Animal Health Faculty of Veterinary Medicine Utrecht University Utrecht The Netherlands; ^2^ Laboratory of Microbiology Wageningen University & Research Wageningen The Netherlands; ^3^ Cargill Animal Nutrition and Health Innovation Center Velddriel Velddriel The Netherlands; ^4^ Department of Population Health Sciences Institute for Risk Assessment Sciences Faculty of Veterinary Medicine Utrecht University Utrecht The Netherlands

**Keywords:** 16S rRNA gene, cecal microbiota, field study, phenotypes, poultry

## Abstract

Disturbances in intestinal health are a common problem affecting commercial broiler chickens worldwide. Several studies have revealed associations between health, production performance, and intestinal microbiota. This study aimed to describe the development of the intestinal microbiota of broilers within a production cycle to evaluate to what extent clinical parameters and phenotypic characteristics can explain the intestinal microbiota variation. Of four well‐performing flocks within two farms, the cecal content was collected of nine broilers at 0, 2, 4, or 5, 7, 11, or 12, 14, 21, 28, 35, and 40 days of the production cycle. In total, 342 samples were analyzed using 16S ribosomal RNA gene amplicon sequencing. Variables as macroscopic gut abnormalities, gut lesions, age, individual body weight, sex, footpad integrity, the color of ceca, and foam in cecal content were determined. Ileum tissue was collected for histological quantification of villus length and crypt depth. Flock infection levels of the intestinal disease coccidiosis were measured in pooled feces from the poultry house. Increases in phylogenetic diversity were observed from hatch until day 21 of age. Constrained multivariate analysis indicated that age, farm, body weight, ileum crypt depth, cecal color, and the coccidiosis lesion score were important variables to describe the variation in cecal microbiota. These results contribute to determining relevant variables in flocks that may be indicative of the intestinal microbiota composition. Moreover, this knowledge increases the awareness of interactions between the intestinal microbiota and broiler health as well as their relative importance.

## Graphical abstract

The microbiota composition in the ceca of broiler chickens developed strongly in the first week of life, but the phylogenetic diversity stabilized around 21 days of age. The variation in cecal microbiota composition in well‐performing broiler flocks under field conditions was mostly explained by age, farm, broiler body weight, ileum crypt depth, the color of the cecal content, and coccidiosis lesion scores. This study contributes to the understanding of the development and the interaction between intestinal microbiota and broiler health.

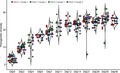

## 1 INTRODUCTION

Disturbances in intestinal health in broiler chickens are a common worldwide challenge (M'Sadeq, Wu, Swick, & Choct, 2015). Intestinal problems reported in broilers are often associated with a microbial imbalance, defined as a deviation from the composition observed in a healthy state, regularly referred to with the ill‐defined terms “dysbiosis” or “dysbacteriosis” (Ducatelle et al., 2018; Teirlynck et al., 2011). Intestinal health problems have been associated with a loss in production performance, and several studies have correlated this with intestinal microbiota composition (Han et al., 2016; Hofshagen & Kaldhusdal, 1992; Johnson et al., 2018). Therefore, knowledge of the development and variation of intestinal microbiota composition is pivotal for the design of strategies toward optimizing the intestinal health of broiler flocks. In poultry, it has been observed that the gut microbiota composition varies between flocks, flock cycles, breeds, and housing conditions and that many other unknown host and environmental factors exist (Cuperus, Kraaij, Zomer, van Dijk, & Haagsman, 2018; Johnson et al., 2018; Kers, Velkers, et al., 2019; Kers et al., 2018; Kim et al., 2015; Stanley, Geier, Hughes, Denman, & Moore, 2013).

Currently, it is unclear which phenotypic characteristics and clinical parameters are important to be able to differentiate and describe the intestinal microbiota composition associated with either healthy or unhealthy humans and animals. In humans, for example, the self‐assessed Bristol stool scale score to assess stool consistency was identified as an important phenotypic factor that showed the largest effect size to explain the variation in fecal microbiota composition (Falony et al., 2016). Food is also known to have an impact on microbiota composition, but similar foods can have different effects on people's microbiota (Johnson et al., 2019). In chickens, the use of antibiotics, feed composition, and feed additives are well‐known factors that influence intestinal microbiota composition (Burel & Valat, 2009; Gao et al., 2017; Torok, Allison, Percy, Ophel‐Keller, & Hughes, 2011; Wei, Morrison, & Yu, 2013); however, knowledge concerning factors related to intestinal health is limited.

For the evaluation of intestinal health, two macroscopic scoring systems are commonly used in broiler flocks. The first one, the coccidiosis lesion score (CLS), is used to determine the presence and severity of infections with *Eimeria* species, causing coccidiosis (Johnson & Reid, 1970). The second is a macroscopic score to assess the severity of intestinal health problems, ranging from a subclinical microbial imbalance to enteritis. This score was developed and introduced as a morphometric evaluation of "dysbacteriosis," further referred to as the gut score (GS) (Teirlynck et al., 2011). It has also been described that a normal cecal lobe contains dark free‐flowing fecal material and that pale ceca with dense non‐free‐flowing content have been associated with inflammation (Atterbury et al., 2011). These observations have, however, not yet been studied in association with cecal microbiota composition.

Knowledge of the dynamics in the development of the cecal microbiota is important, as it will help to optimize the timing of interventions. However, in literature, there is still a lack of consensus on the age of the maturation or stabilization of the cecal microbiota of broiler chickens. One study showed that the cecal content microbial community resulted in no differences between 14 and 28 days of age (Lu et al., 2003). This is in contrast with a study that showed higher individual variability in cecal microbiota of broilers of 14 days versus 28 days of age, suggesting that the cecal microbiota is not yet stable in 14‐day‐old broilers (Torok, Hughes, Ophel‐Keller, Ali, & Macalpine, 2009). Another study showed no differences in cecal microbiota composition in broilers between day 22 and day 36 of age (Ranjitkar, Lawley, Tannock, & Engberg, 2016).

The purpose of the present study was therefore to describe the development of the intestinal microbiota composition of broiler chickens within a production cycle from different flocks and to evaluate whether and to what extent phenotypic characteristics and clinical parameters can explain the observed microbial variation. Two commonly used clinical scoring systems, the CLS and GS (Johnson & Reid, 1970; Teirlynck et al., 2011), were used to assess possible correlations between health status and cecal microbiota composition. Furthermore, phenotypic characteristics, such as broiler age, individual body weight, sex, footpad integrity, and color of the ceca and foam in cecal content, were measured. At poultry house level, the number of *Eimeria* oocysts per gram was measured in pooled feces at days 14, 21, 28, and 35 to determine flock infection levels of the intestinal disease coccidiosis. The microbiota composition of cecal content was assessed by 16S ribosomal RNA (rRNA) gene amplicon sequencing. Also, ileum tissue was collected to measure villus length and crypt depth. In total, the data of 342 broilers, and 14 phenotypic characteristics and clinical parameters as explanatory variables, were included in multivariate distance‐based redundancy analysis to study associations with intestinal microbial development.

## 2 MATERIALS AND METHODS

### 2.1 Farm selection

**FIGURE 1:**
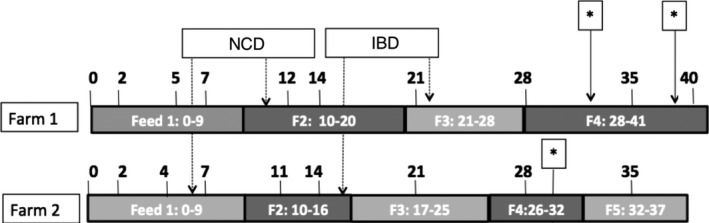
Schematic outline of sampling days and the different feed (F) and vaccination schedules at the two farms included in this study. Both farms have their feed change schedule. This overview shows different vaccination and sampling moments at the farms. NCD = vaccine against Newcastle disease, IBD = vaccine against infectious bursal disease. *Thinning, that is, 15%–30% of broilers are transported to the slaughterhouse

Data for this study were obtained from two broiler farms in the Netherlands, both with Ross 308 broilers. The farms were selected for good production performance, as we were interested in a healthy intestinal microbiota. Also, to reduce the chance of including flocks treated with antibiotics, only farms with antimicrobial use in the previous months below 15 DDDAf (defined daily dose per animal year on farm level) were recruited for the study. Two poultry houses were selected within a farm. They were equal regarding size, year of construction, heating system, cleaning protocols, and feed and water system. The farms received chickens from different commercial hatcheries. The age of the breeder stock was 55 weeks for Farm 1 and 35 weeks for Farm 2. At both farms, water and feed were supplied *ad libitum*. The two farms obtained feed from different suppliers, with small differences in feed composition. The diets on both farms were wheat‐based and were combined with the addition of whole wheat at the farm at later ages. In addition to soybean meal, sunflower seed meal and rapeseed meal were added at a maximum of 6.5% inclusion. Farm 1 started with a bird density of 21 birds/m^2^, and Farm 2 started with 24 birds/m^2^. Figure 1 provides an overview of the different feed changes, vaccination, and flock thinning moments during the production cycle. Farm 1 used wood shavings as litter material, and Farm 2 peat. In both farms, artificial lighting was set to 23 hours/day (h/d) for days 0–3, 20 h/d for days 4–6, and 18 h/d for days 7–35. The temperature was set to gradually decrease from 34°C at day 0 to 20°C from day 35 onwards. Both farms used a combination of the coccidiostatic drugs nicarbazin and narasin (Maxiban^®^ G160, Elanco, Houten, The Netherlands). Farm 1 used nicarbazin and narasin from day 0 until day 28 followed by narasin (Monteban^®^ G100, Elanco, Houten, The Netherlands) until the end of the production period. Farm 2 used nicarbazin and narasin from day 0 until the end of the production period. No other antimicrobial treatments were applied during the study.

### 2.2 Data collection

**FIGURE 2:**
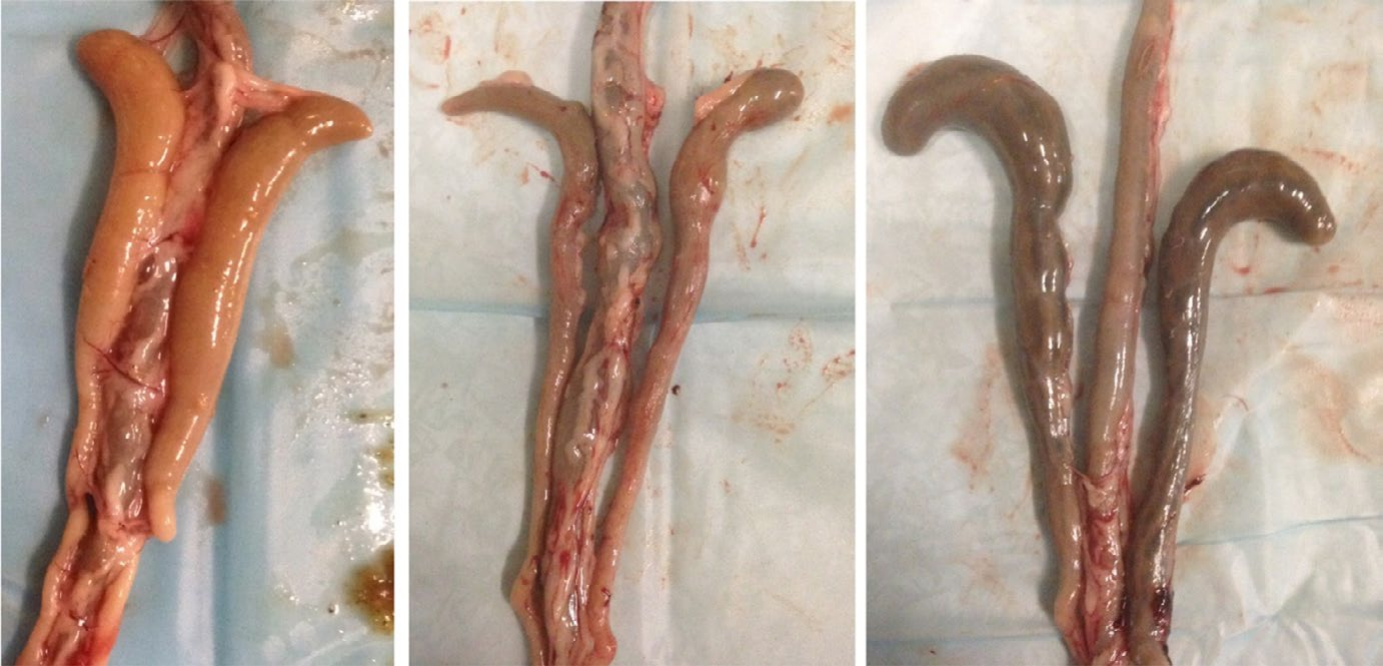
Color classification of cecal lobes. Three different colors of ceca. (a) light content, with foam; (b) dark content, no stripes, and no foam; (c) dark content, with stripes and no foam

During the production cycle Farm 1 was visited on days 0 (day of chick placement), 2, 5, 7, 12, 14, 21, 28, 35, and 40 (August 2016), and Farm 2 on days 0, 2, 4, 7, 11, 14, 21, 28, and 35 (June 2017). From each poultry house, nine broilers were randomly selected for sampling at each of the visits. Between the sampling of the two poultry houses on the same farm, coveralls, footwear, and all sampling materials were changed. The start of the sampling of broilers took place at least 30 min after the end of a dark period, to avoid low amounts of content in the intestinal tract at sampling. The broilers were individually weighed and checked for external abnormalities, including evaluation of absence or presence of footpad abnormalities. Footpad integrity, such as discolorations or lesions, was scored according to methods developed for broiler welfare legislation (Broiler Directive 2007/43/EC), with a score of 2 or above considered as the presence of footpad abnormalities (Welfare Quality, 2009). The broilers were euthanized by cervical dislocation. The gastrointestinal tract was quickly but carefully removed, and this procedure was carried out as much as possible in a sterile fashion, as previously described in detail (Kers, Fischer, Stegeman, Smidt, & Velkers, 2019). The cecal lobes were classified into three classes based on external visual inspection: light color of the cecal content (A), dark content without stripes (B), and dark content with stripes (C) (Figure 2). Also, the presence of foam in the cecal content was recorded. The distal dead end of the cecum was cut to collect cecal content. The cecal content was gently squeezed into a 2‐ml sterile cryotube, snap‐frozen on dry ice, and stored at −80°C until further use for DNA extraction. The time between euthanization and placing the cecal samples on dry ice ranged between 3 and 5 min. After the collection of cecal content, the gonads were inspected to determine the sex of the broiler. Between broilers, sterile gloves were changed, and the table, scissors, and tweezers were cleaned with 70% ethanol to prevent cross‐contamination between broilers.

On days 14, 21, 28, and 35, the intestinal tract was scored macroscopically using two methods. We quantified intestinal mucosa lesions indicative for *Eimeria* spp. infections, CLS (Johnson & Reid, 1970), and we applied the macroscopic “dysbacteriosis” score system, GS (Teirlynck et al., 2011). The CLS was quantified on a scale from 0 to 4, with score 0 when lesions were absent and score 4 to indicate the most severe lesions. This score was determined for three separate parts of the intestinal tract, corresponding with the multiplication sites of the three most common *Eimeria* spp. in broilers: the duodenum (*E*.* acervulina*), jejunum (*E*.*maxima*), and ceca (*E*.*tenella*). The total CLS score ranged from 0, no lesions, to 12, severe lesions in multiple sites in the intestinal tract. The GS consisted of 10 parameters that were assessed using a binary system, with or without the presence of (1) ballooning of the gut; (2) significant redness or dilated blood vessels cranial or (3) caudal to Meckel's diverticulum; (4) reduced gut wall thickness or increased fragility of the gut cranial or (5) caudal to Meckel's diverticulum; (6) reduced tonus (flaccidness) of the gut cranial or (7) caudal to Meckel's diverticulum; (8) abnormal appearance of the contents in the lumen of the gut cranial or (9) caudal to Meckel's diverticulum; and (10) undigested feed particles caudal to the ileocecal junction (Teirlynck et al., 2011). The GS score was 0, when no gut abnormalities were found, and ranged to 10, indicating the presence of all 10 gut abnormalities included in the scoring system. Per time point, CLS and GS data were collected from nine broilers from Farm 1 and 18 broilers from Farm 2.

On days 14, 21, 28, and 35, the number of oocysts per gram of feces (OPG) was quantified in a pooled fecal sample collected from the litter. A modification of a McMaster oocyst counting chamber technique was used as previously described (Velkers et al., 2010). As the McMaster technique is not suitable for reliable species identification, and each *Eimeria* species might have a specific impact on microbiota composition, a quantitative polymerase chain reaction (qPCR) for seven chicken *Eimeria* spp. (*E*.*acervulina*,*E*.*maxima*, *E*.*tenella*, *E*.*mitis*, *E*.*brunetti*, *E*.*necatrix*, and *E*.*praecox*) was performed on day 35 of the production cycle for species identification (Peek, Ter Veen, Dijkman, & Landman, 2017).

### 2.3 Immunohistochemistry

To study the development of the gut, villus length and crypt depth were measured. Jejunum segments were taken near Meckel's diverticulum from nine broilers per farm on each sampling day. Samples were fixed in formalin, dehydrated in xylene, embedded in paraffin, and sectioned in 5‐μm‐thick slices. The tissue was then stained with hematoxylin and eosin and examined under a light microscope (Olympus, Tokyo, Japan). Representative images were taken, and crypt length and villus length were measured employing image software (Cellsense^®^). For each broiler, measurements were done blinded in 30 well‐oriented villus and crypts. Villus height was measured from the tip of the villus to the base of an adjacent crypt. Crypt depth was measured from the villus–crypt axis to the base of the specific crypt.

### 2.4 Cecal content collection and 16S rRNA gene amplicon sequence analysis

DNA extraction, 16S rRNA gene‐targeted PCR, and analysis of microbiota composition were done as previously described (Kers, Velkers, et al., 2019). Briefly, DNA was extracted from 0.25 g cecal content, using 700 μl of Stool Transport and Recovery (STAR) buffer (Roche Diagnostics Nederland BV, the Netherlands). All 342 cecal samples were transferred to sterile screw‐capped 2‐ml tubes (BIOplastics BV, the Netherlands), used for bead beating. The DNA concentrations were measured with a NanoDrop ND 1000 spectrophotometer (NanoDrop^®^ Technologies, USA), and the DNA samples were stored at −20°C until further use. Barcoded primers covering the variable regions V5‐V6, amplified with 784F and 1064R of the 16S rRNA gene, were used for microbial composition profiling (Ramiro‐Garcia et al., 2016). To ensure high‐quality sequencing data, synthetic mock communities of known composition were used as positive controls (Ramiro‐Garcia et al., 2016) and nuclease‐free water as negative controls. The sequencing of resulting libraries was performed by GATC Biotech (now part of Eurofins Genomics Germany GmbH, Konstanz, Germany) on an Illumina Hiseq 2500 instrument. The 16S rRNA gene amplicon data were analyzed using NG‐Tax 2.0 (Poncheewin et al., 2020; Ramiro‐Garcia et al., 2016). In short, to generate amplicon sequence variants (ASVs), NG‐Tax 2.0 employs a fast de novo ASV‐picking algorithm. To assign taxonomy, the SILVA 128 16S rRNA gene reference database was used (Quast et al., 2013).

### 2.5 Statistical analysis

All statistical analyses were performed in R version 3.4.2 (R Foundation for Statistical Computing, Austria), using the packages: Phyloseq, Microbiome, Vegan, and nlme (Lahti, Shetty, Blake, & Salojarvi, 2017; McMurdie & Holmes, 2013; Oksanen et al., 2019; Pinheiro, DebRoy, & Sarkar, 2018). To test for differences in the relative abundance of genera between two groups, we used a Wilcoxon rank‐sum test and corrected for multiple testing with Benjamini–Hochberg (BH) (*q*‐value) and values of *q* < 0.05 were considered significant. Alpha diversity (within‐sample) was determined using phylogenetic diversity (Faith, 2006). Beta diversity (between samples) was determined using Bray–Curtis, Jaccard, and weighted and unweighted UniFrac distance metrics (Bray & Curtis, 1957; Jaccard, 1912; Lozupone, Hamady, Kelley, & Knight, 2007). Differences in alpha diversity between treatment groups were tested with a Kruskal–Wallis test, and pairwise comparisons were tested using a Wilcoxon rank‐sum test. Multivariate microbiota data were visualized using principal coordinate analysis (PCoA), and non‐parametric permutational analysis of variance (PERMANOVA) tests, to analyze group differences within multivariate community data (Anderson, 2001). PERMANOVA was performed with 9999 permutations. To examine differences in total microbiota composition between poultry houses over time, a principal response curve (PRC) analysis was performed. PRC was originally developed to analyze time‐series data and carries out partial redundancy analysis (RDA) ordination to obtain estimates of community changes using time as a predictor variable (Van den Brink & Braak, 1999). Also, distance‐based unweighted UniFrac (UF) and weighted UniFrac redundancy analysis (WUF‐db‐RDA), a multivariate canonical ordination analysis method that considers the phylogenetic makeup of microbial communities, was performed using ASV‐level data (Shankar, Agans, & Paliy, 2017). To determine the most parsimonious constrained ordination model, a stepwise selection (both directions) was used based on the Akaike information criterion (AIC) selection, and a variance inflation factor (VIF) was used to verify the absence of multicollinearity.

## 3 RESULTS

### 3.1 Performance of the flocks and health status

**FIGURE A1:**
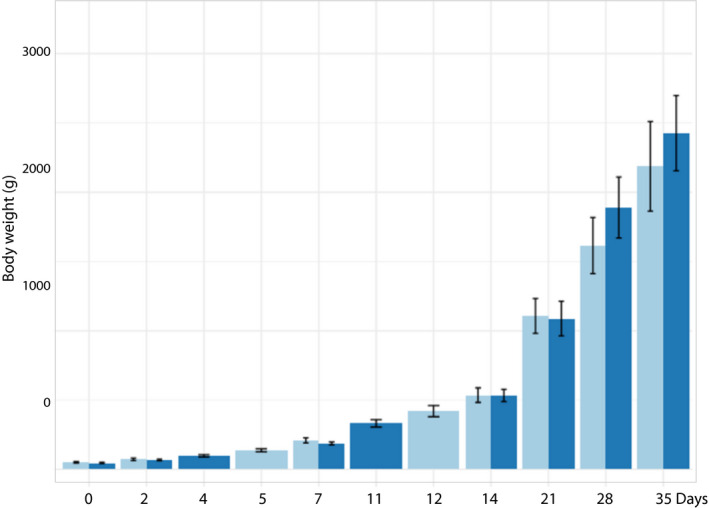
Body weight of broilers across time between farms. No statistical difference in performance was observed between the farms (Farm 1 dark blue, Farm 2 light blue). In both farms, the body weight was above the Ross 308 performance standards (Aviagen, 2019)

Based on the body weight and feed conversion ratios, all flocks were considered as well‐performing compared with Ross 308 broiler production standards (Table A1 in Appendix 1). Bodyweight of the broilers was comparable between farms and poultry houses on the same farm (Figure A1). The oocyst counts indicated some differences in *Eimeria* flock infection dynamics between flocks on days 21, 28, and 35, but in all cases, counts were indicative of rather standard levels of infection on broiler farms (Table A1). On both farms, the *Eimeria* qPCR at day 35 revealed only the presence of *E*.*acervulina*. The condemnation rate at slaughter was slightly above (Farm 2) or considerably lower (Farm 1) than average for European broiler farms. All these findings were in line with the good production performance of both farms and clinical observations during farm visits.

### 3.2 Intestinal microbiota development

**FIGURE 3:**
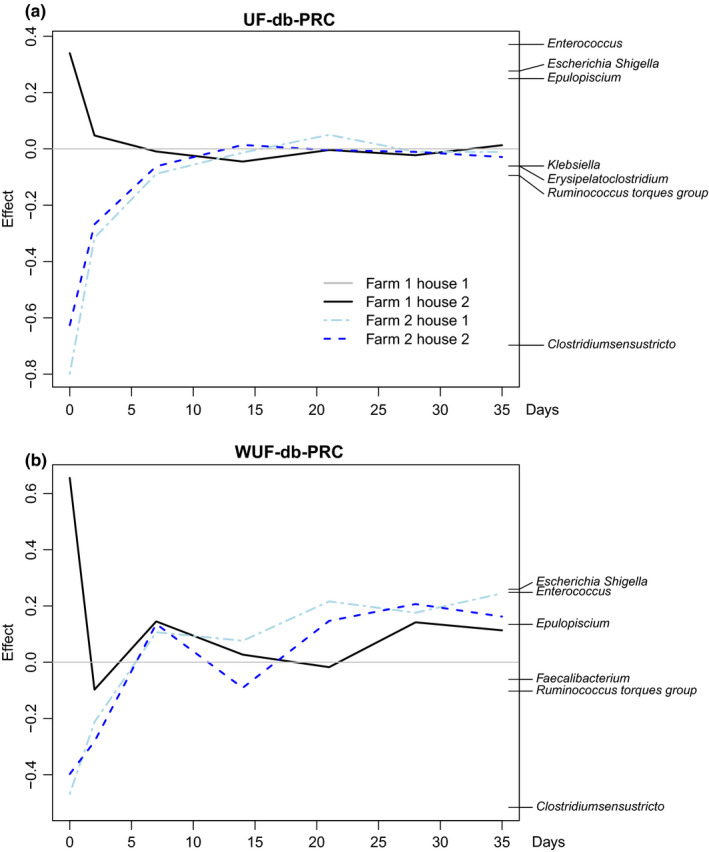
Differences in temporal dynamics of cecal microbiota composition between farms and houses. Principal response curve (PRC) analysis based on unweighted (a) and weighted (b) UniFrac distances between samples at the genus level. Genus weights contributing with an effect above 0.1 to each statistical model are shown on the right side of each panel. Each curve corresponds to one of the poultry houses, with Farm 1, poultry house 1, set as a reference baseline. For each house and time point, data from nine individual broilers are included

Potential differences in the dynamic development of cecal microbiota composition between farms and houses were first globally assessed using PRC analysis based on UF and WUF distances between individual samples and using Farm 1, poultry house 1, as a reference poultry house (Figure 3). Differences in microbial composition between poultry houses were highest on day 0 (day of chick placement) and decreased over time. Based on UF‐db‐PRC, the main difference between Farm 1 and Farm 2 was the higher relative abundance of *Clostridium* sensu stricto in Farm 2, and the lower relative abundance of the genera *Escherichia*,*Shigella*, *Enterococcus*, and *Epulopiscium* in Farm 1 (Figure 3a). After day 7 and until the end of the study, the variation between the poultry houses was limited. Observed differences in temporal development in the different farms were more extensive when using WUF distance data but similar in terms of the genera associated with these differences (Figure 3b).

### 3.3 Diversity analysis of cecal microbiota

**FIGURE 4:**
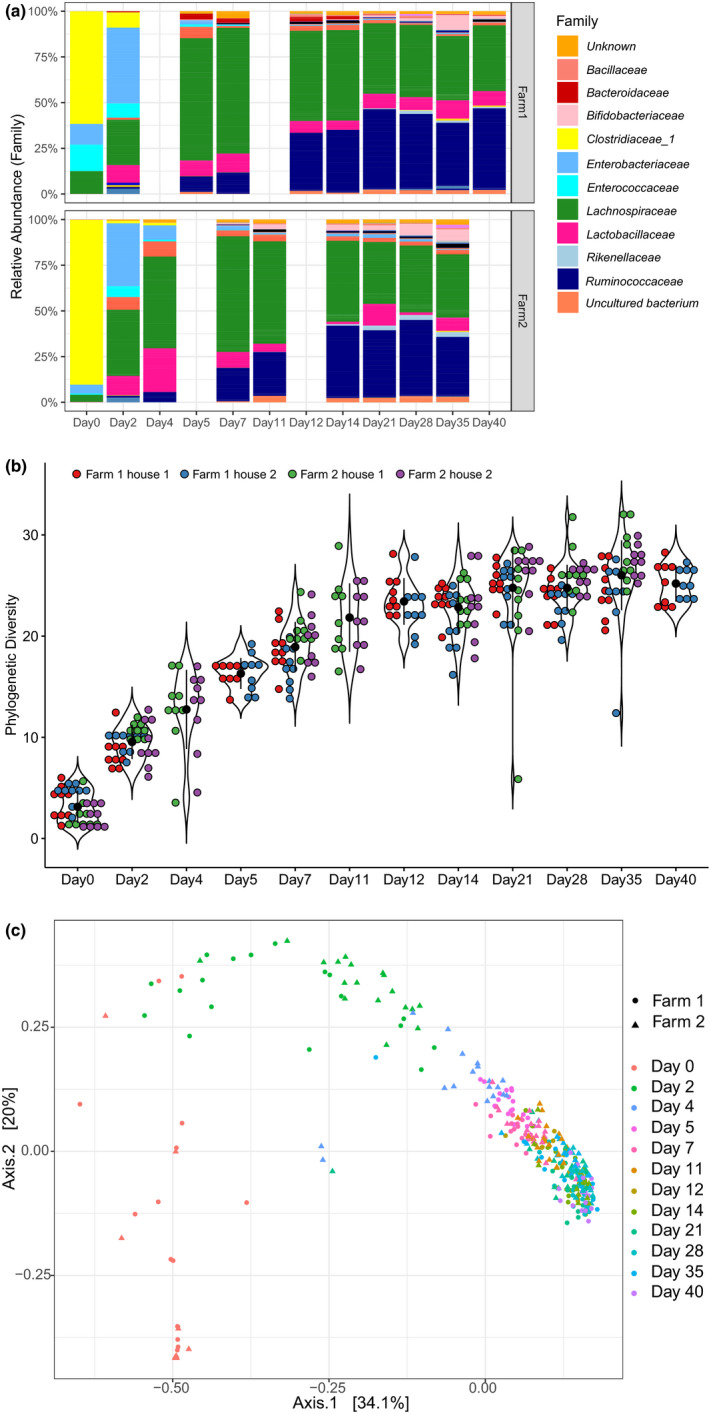
Development of cecal microbiota composition. (a) Development of cecal microbiota in Farm 1 (top panel) and Farm 2 (bottom panel) at the family level. For each time point, data from 18 animals were included (9 animals per house). (b) Phylogenetic diversity per flock increased with broiler age (*n* = 342) until day 21 (pairwise Wilcoxon rank‐sum test, adjusted *p*‐value of *q* < .05). (c) Analysis of beta diversity using principal coordinate analysis based on weighted UniFrac distances. Farm and sampling time are indicated as variables

Overall, in both farms, members of the family *Clostridiaceae* predominated the cecal microbial community on day 0 (Figure 4a). On day 2, *Enterobacteriaceae* and *Lachnospiraceae* were most predominant, whereas members of the *Lachnospiraceae* were predominant on day 4 until day 7. From day 14 until day 40, *Ruminococcaceae* and *Lachnospiraceae* were the most predominant families.

The phylogenetic diversity (alpha) increased with broiler age in both farms until day 21 (Figure 4b). After day 21, however, no significant difference in phylogenetic diversity was found between days, indicating stabilization of microbiota complexity. Although both farms showed the same trend, the phylogenetic diversity was higher in Farm 2 compared with Farm 1 (chi‐squared = 4.62, *df* = 1, *p*‐value = 0.032). When stratified per day, however, significant differences between farms were only found for days 0, 2, 7, 28, and 35, but not for days 14 and 21 (Table A2).

PERMANOVA of cecal microbiota compositional data using WUF showed that age explained 18.6%, whereas farm explained only 1.2% of the observed microbiota variation between broilers, in line with the grouping observed in the PCoA (Figure 4c, Table A3). Depending on whether the analysis was based on UF or WUF distance measures, the difference between farms was highest at day 0 or day 2, in line with the corresponding UF‐ and WUF‐db‐PRC results (Figure 3). In contrast, poultry house did not significantly contribute to explaining the observed variation, and neither did sex, whereas cecal color and foam explained 5.5% and 3.4% of the variation, respectively (WUF, Table A3).

Only on days 21, 28, and 35, the clinical parameters to assess intestinal health, that is, GS and total CLS scores, showed values above zero. The highest average total CLS was found on day 28 and for GS on day 35, but both scores were rather low with the highest value of 5 (Table A4). Therefore, the effect of the GS and total CLS on the microbiota variation between broilers was only tested in a subset of the data for days 21, 28, and 35. The GS did not significantly contribute to explaining the variation, but the total CLS explained 12.4% of the variation (WUF, Table A3).

### 3.4 Factors that explain the intestinal microbiota variation of broilers

**FIGURE 5:**
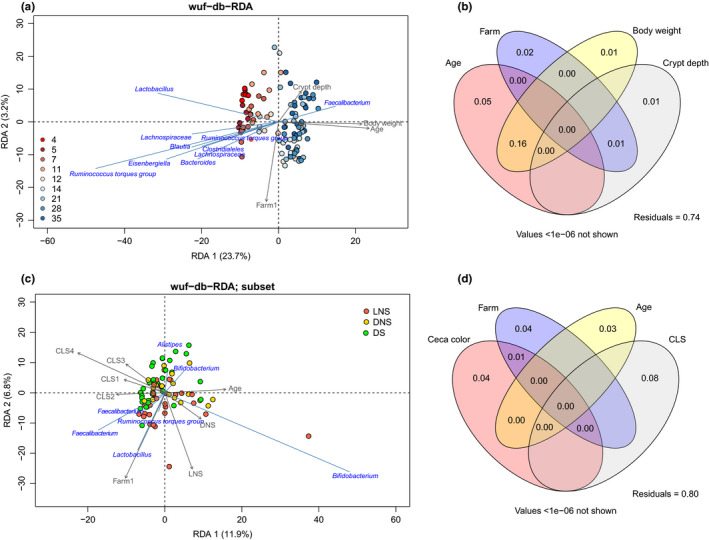
Weighted UniFrac distance‐based RDA and variation partitioning. (a) Triplot for weighted UniFrac distance‐based redundancy analysis (WUF‐db‐RDA) of cecal microbiota composition on the ASV level. The longer the arrow, the stronger the correlation. Samples are colored by age (*n* = 111 broilers), and taxonomic labels are on the lowest know level. (b) Venn diagram visualizing the variation partitioning of WUF‐db‐RDA and showing a strong overlap between age and body weight (16%). (c) Triplot WUF‐db‐RDA of subset broilers with ages 21, 28, and 35. In this dataset, only cecal color, farm, age, and CLS are significant (*n* = 81 broilers). Points are colored by cecal color (LNS, light without stripes; DNS, dark without stripes; DS, dark with stripes; no light‐colored ceca with stripes were observed). (d) Venn diagram visualizing the variation partitioning of the WUF‐db‐RDA subset and showing no overlap between the explanatory variables

**FIGURE A2:**
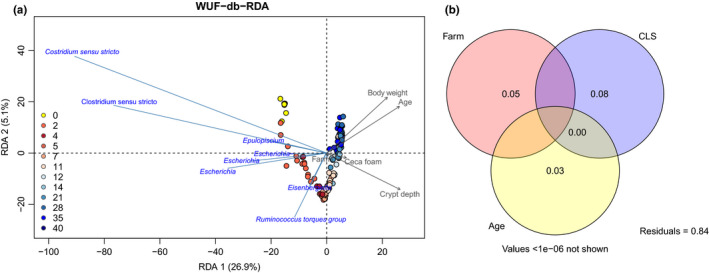
Weighted UniFrac‐db‐RDA of cecal microbiota composition. riplot for weighted UniFrac‐db redundancy analysis (WUF‐db‐RDA) of cecal microbiota composition. The longer the arrow, the stronger the correlation. (a) All time points included in the model. (b) The Venn diagram displaying the variation partitioning of the WUF‐db‐RDA subset showed no overlap between the explanatory variables farm, coccidiosis lesion scores (CLS), and age (*n* = 148 broilers)

To further disentangle the different effects of phenotypic characteristics and clinical parameters on cecal microbiota composition, WUF‐db‐RDA was carried out using data at the ASV level. The first model contained 148 broilers of both farms of which all 11 phenotypic characteristics were measured (Table A5). The most parsimonious model to explain the variation of the cecal microbiota included five significant explanatory variables, that is, crypt depth, age, body weight, cecal foam, and farm, and explained 34.8% of the cecal microbiota variation. The variation in the relative abundance of members of the genus *Clostridium* sensu stricto, strongly predominant on days 0 and 2, was most accurately accounted for by the model (Figure A2). Therefore, those days were removed in the next model. The most parsimonious WUF‐db‐RDA model based on 111 broilers contained the four significant explanatory variables age, farm, body weight, and crypt depth and explained 28.9% of the variation (Figure 5a). The relative abundance of a member of the genus *Faecalibacterium* was associated with the ordination in broilers with older age, higher body weight, and crypt depth (Figure 5a). Venn diagrams, visualizing the partitioning of variation of WUF‐db‐RDA and shared variance explained by the significant explanatory variables, showed a strong overlap between age and body weight among the explanatory variables tested, accounting for the largest explained variance (16%, VIF 17) (Figure 5b). Also, the four significant explanatory variables explained part of the variation independently, that is, age (5%), farm (2%), body weight (1%), and crypt depth (1%) (Figure 5b).

The final model contained only data of days 21, 28, and 35 and included the clinical parameters (log10 OPG, CLS, and GS), and the previously described significant explanatory phenotypic variables age, farm, body weight, and cecal color (no crypt depth data were available for these broilers). The most parsimonious model to explain the variation in this dataset included cecal color, farm, age, and CLS, and explained 28.5% of the cecal microbiota variation (Figure 5c). The analysis of variance partitioning among the explanatory variables tested showed only a 1% overlap between farm and cecal color. The explanatory variables that individually explained part of the variation were CLS (8%), farm (4%), cecal color (4%), and age (3%) (Figure 5d). CLS was associated with a higher relative abundance of a member of the genus *Bifidobacterium* (Figure 5c).

## 4 DISCUSSION

This comprehensive study described the dynamics of the development of the cecal microbiota for four different broiler flocks that were frequently sampled throughout a production cycle. Furthermore, the variation in cecal microbiota composition was associated with phenotypic characteristics and clinical parameters of broilers chickens.

Although the mechanisms driving cecal microbiota development remain elusive, these data suggest that the development is major in the first week of life and that after day 21 cecal microbiota composition can be considered as mature and stable in a commercial well‐performing broiler flock. Our finding that age had the most profound effect on the cecal microbiota is in line with the literature (Awad et al., 2016; Gong et al., 2008; Wielen, Keuzenkamp, Lipman, van Knapen, & Biesterveld, 2002). A small number of studies used day‐old chicks of different avian species (Ballou et al., 2016; Smith & Rehberger, 2018). Those studies showed that the phylum *Proteobacteria* was predominant on day 0 (Ballou et al., 2016; Smith & Rehberger, 2018), although in turkeys also the family *Clostridiaceae* was observed to be predominant on day 0 (Smith & Rehberger, 2018), similarly to our study. In 3‐day‐old laying hen pullets, also *Clostridiaceae* and *Enterobacteriaceae* were found to be highly abundant (Mon et al., 2015). *Lachnospiraceae* became predominant from day 8 onwards (Mon et al., 2015), and *Lactobacillaceae* were not observed in day‐old chicks but were observed in chicks from 3 days of age onwards (Gong et al., 2008), which is in line with our observations. Our study showed no difference in genera between days 21 and 28, and phylogenetic diversity seemed to stabilize after 21 days, despite feed changes that occurred on both farms after 21 days. These results indicate the maturation of the cecal microbiota, with no large developmental changes afterward.

Studies on the dynamics of the development of the microbiota of children have shown that in the first months of life *Proteobacteria* are predominant. At the age of one year, the genus *Bifidobacterium* is highly abundant, and at the age of two years, members of the class *Clostridia* are among the predominant taxa (Korpela & de Vos, 2018). Although this succession in early life has been based on the five most predominant bacterial taxa of healthy vaginally born children, they showed overlap with the development observed in this broiler chicken study. On day 2, *Enterobacteriaceae* (*Proteobacteria)* were predominant in the cecal content of both farms. After day 7, *Ruminococcaceae* and *Lachnospiraceae* (both *Clostridia*) showed the highest relative abundance in the cecal content. However, in this study, on day 0 the cecal content was predominated by *Clostridiaceae*, a family of mainly obligate anaerobes. This is notable because in human research it is suggested that the first colonizers of the gut are predominantly facultative anaerobes and that later in life obligately anaerobic bacteria take over (Bäckhed et al., 2015; Nagpal et al., 2016). In children born through cesarean section, also a higher relative abundance of obligately anaerobic taxa and lower abundance of facultatively anaerobic taxa have been observed (Nagpal et al., 2016). As commercial chickens hatch under strict hygiene practices in hatcheries, they are exposed to a diverse range of microorganisms from environmental sources rather than from their parents, which might explain the predominance of an obligately anaerobic genus as *Clostridium* sensu stricto (*Clostridiaceae*). This predominance of the genus *Clostridium* has also been reported in day‐old turkeys on a commercial farm (Smith & Rehberger, 2018). Nevertheless, probably not all members of the genus *Clostridium* are truly obligately anaerobic (Wiegel, Tanner, & Rainey, 2006). Although they cannot use oxygen as the terminal electron acceptor, many members of this genus can tolerate oxygen, especially under non‐growth conditions, that is, in the absence of utilizable substrates and energy sources (Wiegel et al., 2006). This suggests that members of the genus *Clostridium* sensu stricto are among the first colonizers of the avian ceca in the field setting studied here and that after a few hours/days members of the phylum *Proteobacteria* colonize the ceca, followed later in life by other members of the *Clostridiaceae*. Members of the family *Clostridiaceae* are known as spore‐forming bacteria (Wiegel et al., 2006). This might be how they find their way into the egg or day‐old‐broilers (Richards‐Rios, Leeming, Fothergill, Bernardeau, & Wigley, 2020).

Explanatory variables as CLS, cecal color, farm, and age were associated with the microbiota variation in broilers of 21 days and older. In the total dataset, age was found to be the most important factor, whereas, within the last three time points (21, 28, 35 days), age was significant but not the most important explanatory variable as the microbiota composition had become more mature and thus stable. Although only a limited number of lesions were observed, it is striking that it was still the most important explanatory variable in the dataset comprising days 21, 28, 35, suggesting that on farms with more severe coccidiosis problems even larger effects may be expected. The CLS was associated with a higher relative abundance of *Bifidobacterium*. This is in line with an experimental study where broilers with an *E*.*tenella* infection, a species multiplying in the ceca, also showed a higher relative abundance of *Bifidobacterium* compared to broilers without infection (Macdonald et al., 2017). Nevertheless, *E*.*tenella* was not observed in the pooled fecal samples by qPCR nor were lesions in the ceca observed at post‐mortem. The species *E*.*acervulina* that multiplies in the duodenum was the only species observed on both farms, which may have caused shifts in the microbiota composition downstream as described previously (Hauck, 2017; Perez et al., 2011).

In humans, medication, stool consistency, and transit time were variables that explained part of the stool microbiota variation (Falony et al., 2016; Müller et al., 2020). In our study, also the color of the ceca was associated with cecal microbiota composition, although no overlap with any of the clinical parameters was observed. In literature, pale ceca, due to white blood cell infiltration, have been associated with infection by, for example, *Salmonella* (Atterbury et al., 2011). We can speculate that the lack of overlap between cecal colors with any of the clinical parameters suggests that in our study the color was not associated with infection but rather by fermentation phase or transit time.

Though the farms included in this study were both well‐performing, farm explained part of the variation, in contrast to the flock. As every flock lived in a different house, the variable flock was expected to influence the microbiota composition as previous research has shown (Kers, Velkers, et al., 2019). Because the flocks in the two poultry houses on each of the farms started with similar exposure to microbes, we can speculate that this similar early‐life exposure to microbes affected and shaped microbiota composition then and later in life.

Previous research using the GS system showed correlations of gut scores measured at days 10, 17, and 20, indicating that the prevalence of gut abnormalities at day 10 can be predictive for scores later (Caekebeke et al., 2020). It is also likely that disruptions in microbiota composition occur before clinical signals, such as production losses, become apparent. In this study, however, the gut score was not associated with cecal microbiota composition. All flocks in this study were well‐performing, and only low CLS and GS values were observed. This may have limited the chance of observing associations between clinical parameters and the intestinal microbiota. This limitation in the current analysis also addresses an important general challenge for these types of studies. A lot of different host‐related factors and infectious and non‐infectious challenges may directly or indirectly affect the broiler microbiota composition and can consequently influence broiler health and performance (Kers et al., 2018). In humans, it has been described that over 500 individuals per group are needed to study shifts in microbiota composition in, for example, obese versus lean people (Falony et al., 2016). In this study, we analyzed nine birds per timepoint per flock. Although the total sample size is larger than in most other poultry studies published to date, this might still not be enough, especially considering that within the same flock, the sampled broilers are not independent of each other. Only a limited number of phenotypic or clinical characteristics were found to be associated with cecal microbiota variation in this study. A larger sample size per flock and a larger number of farms, with more distinct differences in health and performance status, might have revealed more associations between microbiota composition and phenotypic and clinical characteristics.

This study was performed using the V5‐V6 region, and within the used pipeline, it was shown to outperform the V4 region, for example, allowing for higher differentiation within the *Enterobacteriaceae* family and showing great consistency with full‐length analyses (Ramiro‐Garcia et al., 2016). This limited the comparison of our results across literature because the hypervariable regions give specific taxonomic signatures. However, there is no clear consensus on the preferred region within poultry microbiota studies and a wide variation of regions is used.

To further understand the mechanisms that drive the development and to identify important causes of disruptions in the microbiota associated with reduced health, species‐level or even strain‐level information and knowledge of the actual functioning of the microbes will be needed. Species‐level classification for 16S rRNA gene sequences remains a serious challenge, which limits the interpretation of the results. For example, *Clostridium perfringens* has been associated with dysbacteriosis and bacterial enteritis (Roberts et al., 2015; Teirlynck et al., 2011), but based on the used SILVA reference database the species *Clostridium perfringens* is a member of the genus *Clostridium* sensu stricto *1* that is predominant in day‐old broiler chickens without any clinical signs. Approaches providing a higher level of taxonomic and functional resolution will be needed, such as shotgun metagenomics, that allows for differentiation of functionally distinct, but phylogenetically similar populations. However, even with metagenomics, the functional insights are limited to the prediction of functions, but no information is obtained on the actual metabolic activity. Thus, multi‐omics approaches such as a combination of metagenomics with metabolomics are necessary to identify not only the bacteria but also their metabolites that are important for broiler physiology, production, and health. Furthermore, more observational longitudinal studies including farms with poorer production performance and health status are needed. Such studies may, for instance, validate our associations of the higher CLS with a higher relative abundance of *Bifidobacterium* in the microbiota composition and may reveal other associations with clinical data as well.

In conclusion, in this study age, farm, body weight, crypt depth, cecal color, and CLS were important explanatory variables to explain the variation of the cecal microbiota composition in broiler chickens under field conditions. Our results suggest that the temporal development is major in the first week of life and that after 21 days of age the cecal microbiota composition can be considered as mature and stable in a well‐performing broiler flock. This knowledge contributes to the understanding of the development and the interaction between the intestinal microbiota and broiler health.

## CONFLICT OF INTERESTS

None declared.

## AUTHOR CONTRIBUTION

**Jannigje G. Kers:** Conceptualization (supporting); Data curation (lead); Formal analysis (lead); Investigation (lead); Visualization (lead); Writing‐original draft (lead); Writing‐review & editing (equal). **Jean E. de Oliveira:** Conceptualization (supporting); Writing‐review & editing (supporting). **Egil A. J. Fischer:** Conceptualization (supporting); Writing‐review & editing (supporting). **Monique H. G. Tersteeg‐Zijderveld:** Investigation (supporting); Resources (supporting). **Prokopis Konstanti:** Formal analysis (supporting); Investigation (supporting); Writing‐review & editing (supporting). **Jan Arend (Arjan) Stegeman:** Conceptualization (supporting); Funding acquisition (supporting); Writing‐review & editing (supporting). **Hauke Smidt:** Conceptualization (supporting); Funding acquisition (supporting); Writing‐review & editing (supporting). **Francisca C. Velkers:** Conceptualization (lead); Data curation (lead); Funding acquisition (lead); Investigation (supporting); Writing‐review & editing (equal).

## ETHICS STATEMENT

The animal experiment was approved by the Dutch Central Authority for Scientific Procedures on Animals and the Animal Experiments Committee (registration number AVD108002016442) and complied with all relevant legislation.

## Data Availability

The raw sequence data generated during the current study are available in the Sequence Read Archive (SRA) repository at the NCBI under accession PRJNA644193: https://www.ncbi.nlm.nih.gov/bioproject/PRJNA644193

## Appendix 1

**TABLE A1:** Performance across farms

	Farm 1 Flock 1	Farm 1 Flock 2	Farm 2 Flock 1	Farm 2 Flock 2	Ross 308 standards (Aviagen, 2019)
Body weight at slaughter (g)	2819 (D41)	2819 (D41)	2613 (D37)	2643 (D37)	2334 (D37) 2715 (D41)
Feed conversion ratio (FCR)	1.580	1.580	1.505	1.489	1.587 (D37) 1.667 (D41)
Oocyst output (Log10 OPG)	D21 – 2.64 D28 – 4.05 D35 – 5.05	D21 – 3.00 D28 – 4.65 D35 – 4.08	D21 – 3.48 D28 – 4.63 D35 – 4.89	D21 – 4.23 D28 – 4.76 D35 – 3.19	n.a. #1
Condemnation rate at slaughter	0.20%	0.22%	1.38%	1.38%	n.a. #2

Note

#1 Rather standard levels of infection on broiler farms (Peek et al., 2017). #2 For Farm 2, the condemnation rates were slightly above (Farm 2) and below average (Farm 1) compared with average rates for European broiler farms and the reasons for condemnation did not indicate specific abnormalities related to poor health (Löhren, 2012; Salines, Allain, Roul, Magras, & Le Bouquin, 2017) (D = day).

**TABLE A2:** Comparison of phylogenetic diversity between farms at different broiler ages

	Chi‐squared	*p*‐Value
All days	4.62	0.032
Age 0	9.61	0.002
Age 2	4.10	0.042
Age 7	6.57	0.010
Age 14	0.40	0.527
Age 21	0.57	0.448
Age 28	13.47	2.42e−04
Age 35	15.64	7.66e−05

Note

Differences in phylogenetic diversity (alpha diversity) between treatment farms, stratified per age, tested with a Kruskal–Wallis test.

**TABLE A3:** Beta diversity based on different distance metrics

		*n*	Bray–Curtis		Jaccard		UF		WUF	
			*R* ^2^ (%)	*p*‐Value	*R* ^2^ (%)	*p*‐Value	*R* ^2^ (%)	*p*‐Value	*R* ^2^ (%)	*p*‐Value
Age	Farm 1 + 2	341	12.1	1.00e−04***	7.5	1.00e−04***	19.1	1.00e−04***	18.6	1.00e−04***
	Farm 1	179	14.4	1.00e−04***	9.1	1.00e−04***	21.4	1.00e−04***	20.6	1.00e−04***
	Farm 2	162	12.8	1.00e−04***	8.3	1.00e−04***	20.1	1.00e−04***	18.7	1.00e−04***
Age	21 + 28 + 35	107	2.7	1.00e−04***	2.1	1.00e−04***	4.5	1.00e−04***	4.2	1.00e−04***
F1 vs F2	All ages	341	2.0	1.00e−04***	1.5	1.00e−04***	2.0	1.00e−04***	1.2	0.002**
	Age 0	36	24.9	1.00e−04***	23.3	1.00e−04***	19.6	1.00e−04***	11.5	0.013*
	Age 2	36	22.8	1.00e−04***	14.9	1.00e−04***	17.1	1.00e−04***	12.3	0.002**
	Age 7	36	11.1	1.00e−04***	7.7	1.00e−04***	18.4	1.00e−04***	9.7	8.00e−04***
	Age 14	36	6.1	3.00e−04***	4.9	1.00e−04***	13.6	1.00e−04***	7.1	0.026*
	Age 21	35	9.3	1.00e−04***	7.1	1.00e−04***	10.7	1.00e−04***	8.6	3.00e−04***
	Age 28	36	8.3	1.00e−04***	6.3	1.00e−04***	10.9	1.00e−04***	10.7	1.00e−04***
	Age 35	36	10.7	1.00e−04***	7.9	1.00e−04***	13.9	1.00e−04***	8.4	0.003**
	21 + 28 + 35	107	5.2	1.00e−04***	3.7	1.00e−04***	7.0	1.00e−04***	6.2	1.00e−04***
F1; poultry house	All ages	179	1.1	0.018*	1.0	0.009**	0.6	0.299	0.6	0.385
	Age 21 + 28 + 35	53	5.7	1.00e−04***	4.3	1.00e−04***	3.9	0.006**	2.9	0.109
	Age 35	18	8.8	0.048*	7.9	0.040*	6.0	0.376	4.2	0.649
F2; poultry house	All ages	162	0.6	0.387	0.6	0.376	0.4	0.646	0.2	0.931
	Age 21 + 28 + 35	54	2.3	0.170	2.2	0.128	2.2	0.231	1.6	0.597
	Age 35	18	6.1	0.375	6.1	0.338	6.9	0.238	5.2	0.491
Sex	All ages	341	0.2	0.924	0.2	0.920	0.1	0.942	0.1	0.946
Cecal foam	All ages	340	1.9	1.00e−04***	1.3	1.00e−04***	3.8	1.00e−04***	3.4	1.00e−04***
	Age 21 + 28 + 35	107	1.8	0.0033**	1.5	0.002**	1.7	0.010*	2.2	0.009**
Cecal color	All ages	340	3.7	1.00e−04***	2.5	1.00e−04***	5.4	1.00e−04***	5.5	1.00e−04***
	Age 21 + 28 + 35	107	4.0	1.00e−04***	3.1	1.00e−04***	4.8	1.00e−04***	5.6	1.00e−04***
GS	Age 21 + 28 + 35	81	4.7	0.639	4.8	0.665	5.1	0.348	5.1	0.365
CLS	Age 21 + 28 + 35	81	6.2	0.060.	5.6	0.076.	7.2	0.048*	12.4	0.045*
Footpad integrity	Age 21 + 28 + 35	107	1.3	0.064.	1.2	0.048*	1.5	0.015*	1.1	0.290

Note

*R* ^2 ^= Percentage of the variation between chickens explained, *p*‐value = based on PERMANOVA test, GS = gut score (2), CLS = coccidiosis lesion score (18), ****p* < 0.001, ***p* < 0.01, **p* < 0.05. The age of the broilers explains 18.6% of the variation between all the cecal samples, while the farm just explained 1.2% (weighted UniFrac, WUF). When we stratified the data per age, around 10% (7%–12%) of the variation was explained by farm based on weighted UniFrac. Furthermore, unweighted UniFrac distance was slightly higher than the weighted UniFrac distance between the two farms, whereas the opposite trend was observed for corresponding Jaccard and Bray–Curtis distances that are not phylogenetically weighted. Taken together, this indicated that abundant taxa are more phylogenetically related compared with the less abundant taxa between the two farms.

**TABLE A4:** Overview of total CLS and GS scores

		Day 21		Day 28		Day 35
Total CLS	Average (*SD*)	0.19 (0.48)		0.81 (0.78)		0.56 (1.08)
(0–12)	0	85%	0	37%	0	67%
	1 or 2	15%	1, 2 or 3	63%	1,2 or 5	33%
	>3	0%	>3	0%	>6	0%
GS	Average (SD)	2.11 (1.01)		2.37 (1.01)		2.48 (1.09)
(0–10)	1 or 2	59%	1 or 2	63%	1 or 2	48%
	3, 4 or 5	41%	3, 4 or 5	37%	3, 4 or 5	52%
	>6	0%	>6	0%	>6	0%

Note

The coccidiosis lesion score (CLS) is used to determine the presence and severity of infections with *Eimeria* species (Johnson & Reid, 1970) and can range from 0 to 4 with higher scores indicating more severe lesions. CLS was determined for duodenum, jejunum, and ceca. The total CLS is the sum for all three intestinal parts and can range from 0 to 12. The morphometric evaluation of “dysbacteriosis” referred to as the gut score can range from 0 to 10 (GS) (Teirlynck et al., 2011). Average values and standard deviation (*SD*) for total CLS and GS, and the percentage of broilers for three categories of scores are provided for days 21, 28, and 35 of the study.

**TABLE A5:** Phenotypic characteristics and clinical parameters at the flock and individual level

*Phenotypic characteristics*	*Flock level*	*Individual level*
All days	Farm (1 or 2) Flock (1 of 2)	Age (integer) Body weight (numeric) Sex (male or female) Footpad integrity (yes/no) Cecal color (A, B, C) Cecal foam (yes/no) Villus length (numeric) Crypt depth (numeric) Villus/crypt ratio (numeric)
*Clinical parameters*	*Flock level*	*Individual level*
Days 14, 21, 28, and 35	Number of Eimeria oocysts in pooled feces (log10 OPG)	Coccidiosis Lesion Scores (0–12) Gut Score (1–10)
